# The role of CTCF binding sites in the 3′ immunoglobulin heavy chain regulatory region

**DOI:** 10.3389/fgene.2012.00251

**Published:** 2012-11-16

**Authors:** Barbara K. Birshtein

**Affiliations:** Department of Cell Biology, Albert Einstein College of MedicineBronx, NY, USA

**Keywords:** immunoglobulin heavy chain gene locus, enhancers, insulators, CTCF, class switch recombination, Pax5, chromosome conformation capture (3C) assay

## Abstract

The immunoglobulin heavy chain locus undergoes a series of DNA rearrangements and modifications to achieve the construction and expression of individual antibody heavy chain genes in B cells. These events affect variable regions, through VDJ joining and subsequent somatic hypermutation, and constant regions through class switch recombination (CSR). Levels of IgH expression are also regulated during B cell development, resulting in high levels of secreted antibodies from fully differentiated plasma cells. Regulation of these events has been attributed primarily to two *cis*-elements that work from long distances on their target sequences, i.e., an ∼1 kb intronic enhancer, Eμ, located between the V region segments and the most 5′ constant region gene, Cμ; and an ∼40 kb 3′ regulatory region (3′ RR) that is located downstream of the most 3′ C_H_ gene, Cα. The 3′ RR is a candidate for an “end” of B cell-specific regulation of the *Igh* locus. The 3′ RR contains several B cell-specific enhancers associated with DNase I hypersensitive sites (hs1–4), which are essential for CSR and for high levels of IgH expression in plasma cells. Downstream of this enhancer-containing region is a region of high-density CTCF binding sites, which extends through hs5, 6, and 7 and further downstream. CTCF, with its enhancer-blocking activities, has been associated with all mammalian insulators and implicated in multiple chromosomal interactions. Here we address the 3′ RR CTCF-binding region as a potential insulator of the *Igh *locus, an independent regulatory element and a predicted modulator of the activity of 3′ RR enhancers. Using chromosome conformation capture technology, chromatin immunoprecipitation, and genetic approaches, we have found that the 3′ RR with its CTCF-binding region interacts with target sequences in the V_H_, Eμ, and C_H_ regions through DNA looping as regulated by protein binding. This region impacts on B cell-specific *Igh* processes at different stages of B cell development.

## *Igh* GENES AND THEIR DNA REARRANGEMENTS AND MUTATION

The immunoglobulin heavy chain gene locus (*Igh*) undergoes an amazing array of DNA rearrangements and mutagenic events during B cell differentiation (reviewed in Max, 2008). A general question is how these DNA modifications are normally achieved during B cell development without mistakes that result in malignant transformation. Our studies have focused on a regulatory region that acts at long distances on target *Igh* sequences essential for these DNA rearrangement and mutagenic events (reviewed in Pinaud et al., 2011).

The *Igh* locus extends for ∼3 Mb and contains coding segments for constructing a diverse repertoire of variable region genes, through recombination of V_H_ (variable), D_H_ (diversity), and J_H_ (joining) segments, as well as for constant region (C_H_) genes that, when translated, confer different functional capabilities on antibody molecules. During bone marrow B cell development, the locus undergoes sequential DNA rearrangement and mutational events that generate an enormous range of antibody heavy chain genes, each specifying individual antigen binding sites associated with specific constant regions. The initial event, i.e., recombinase-activator genes (RAG)-mediated V(D)J joining, involves first, a DJ join, and then V to DJ joining, both accompanied by deletions of intervening sequences; these lead to expression of a IgM heavy chain bearing a single variable region. Successful expression of one allele halts rearrangements on the other allele (allelic exclusion) and prompts VJ joining on the light chain allele. Upon leaving the bone marrow, the B cell with its H_2_L_2_ surface IgM is poised to receive signals through antigen and other receptors for T cell surface proteins and secreted cytokines that trigger further DNA targeted events, such as class switch recombination (CSR) and somatic hypermutation. CSR is initiated by germline transcription (GT) of the non-IgM C_H_ gene to which subsequent DNA rearrangement will occur. The DNA rearrangement event results in a shift of the VDJ gene segment from its position upstream of μ to upstream of γ, ε or α genes; as in VDJ joining, intervening DNA is deleted as a circle. V_H_-hypermutation results, upon antigen selection, in B cells with higher affinity antigen-binding sites. Both CSR and somatic hypermutation depend on the activity of activation-dependent cytidine deaminase (AID). In fully differentiated plasma cells, heavy chain gene expression occurs at high levels. These multiple processes of VDJ joining, GT and CSR, and increased *Igh* expression levels require tight regulation to contain these potentially mutagenic events within the confines of the *Igh* locus.

## THE 3′ RR CONTAINS AN ENHANCER MODULE AND A HIGH-DENSITY CTCF-BINDING REGION

**FIGURE 1:**
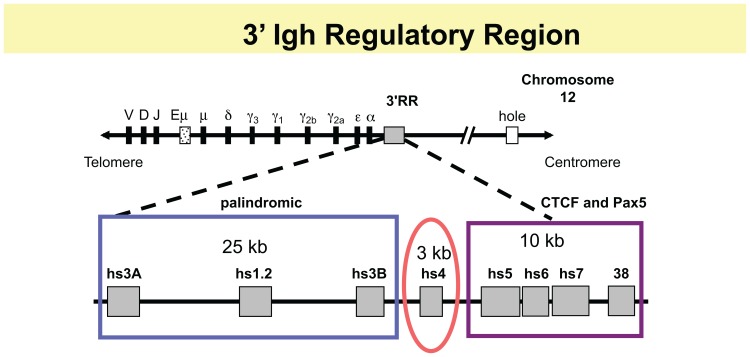
**Schematic of 3′ RR.** The top line shows relative positions of V, D, and J segments, the intronic enhancer, Eμ, and the C_H_ genes. The 3′ RR region is located downstream of the Cα gene of the *Igh* locus and has two major modules: an ∼28 kb region containing four enhancers, that, collectively, are essential for GT and CSR and for high levels of *Igh* expression in plasma cells. The 5′ 3 enhancers, hs3A, hs1.2, and hs3B, occupy a palindromic region (blue box), with hs3A and hs3B in inverted orientation at the ends of the region. A fourth enhancer hs4 occupies a separate structural and functional unit (red oval). In the 10 kb downstream, there is a high-density of CTCF binding sites associated with DNase I hypersensitive sites hs5, hs6, and hs7, and with a segment 4 kb further downstream, termed “38” because it is located ∼38 kb from the beginning of the 3′ RR (with BAC199 M11 as a reference, Genbank AF450245; purple rectangle). This region also contains interspersed Pax5 sites.

Two major long distance *Igh* control elements have been identified. Our focus here is on a large (∼50 kb) 3′ regulatory region (3′ RR), located downstream of the C_H_ genes (reviewed in Pinaud et al., 2011) and schematized in **Figure 1**. A second well-characterized control element is an ∼1 kb intronic enhancer, Eμ, positioned between the V, D, and J segments and the C_H_ genes, which is critical for VDJ joining (reviewed in Max, 2008).The murine 3′ RR contains a 5′ 28 kb segment, which has four enhancers that collectively support GT, CSR, and high levels of IgH expression in plasma cells. An ∼10 kb 3′ segment contains a region of high-density CTCF- and Pax5-binding sites with insulator activity. Pax5, a transcription factor essential for B cell identity (reviewed in Cobaleda et al., 2007), is associated with 3′ RR enhancers as well. Our studies have shown that the 3′ RR interacts at long distances with a number of *Igh* target sites, as part of its influence on CSR and regulation of *Igh* expression. This entire region is a candidate for a downstream “end” of B cell-specific regulation of the *Igh* locus. At the upstream V region end, the *Igh* locus begins in the general vicinity of telomeric sequences (mouse chr. 12, human chr. 14), suggestive of a natural boundary. At the 3′ C_H_-end, beyond the terminus of the 3′ RR, *hole* (*Tmem121*), *Crip1/2*, and *mta1* are the nearest non-*Igh* downstream genes (all in the same inverted transcriptional orientation compared to the *Igh* locus) followed by the rest of the chromosome (Zhou et al., 2002a). There are multiple kinds of regulatory elements in this 3′ RR. Three of the four enhancers located in the 5′ segment of the murine 3′ RR form an ∼25 kb palindrome, in which the central hs1.2 enhancer is flanked by virtually identical terminal enhancers hs3A and hs3B (Saleque et al., 1997). A fourth enhancer, hs4, lies 3′ of hs3B in a separate 3 kb structural and functional unit (Michaelson et al., 1995; Saleque et al., 1997). Hs4 and the palindromic region vary in their acquisition of DNase I hypersensitivity during B cell maturation (Giannini et al., 1993); hs4 becomes hypersensitive early in B cell development and remains so throughout, while the palindromic enhancers become hypersensitive only later in B cell maturation. A similar 3′ RR (hs3, hs1.2, hs4) is located downstream of each of the two Cα genes in the human *Igh* locus (Chen and Birshtein, 1996, 1997; Mills et al., 1997; Sepulveda et al., 2004a,b; Frezza et al., 2009).

**FIGURE 2:**
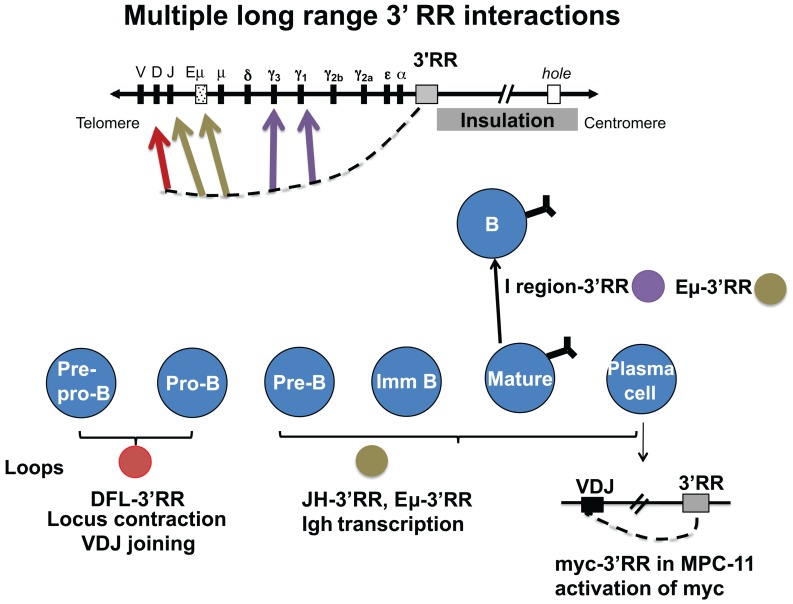
**The 3′ RR interacts via looping with many different target *Igh* sequences during B cell development.** During VDJ joining in pro- and pre-B cells, the 3′ RR interacts with two CTCF sites upstream of D_H_ (red arrow). Also in pre-B cells, the 3′ RR is involved in allelic regulation (brown arrow). In B cells, interaction of the 3′ RR with I/switch regions is associated with GT and subsequent CSR (purple arrows). In plasma cells (lower right), the 3′ RR interacts with the expressed VDJ region and the intronic enhancer. Also in malignant plasma cells, the translocated *c-myc* oncogene interacts in *cis* by looping with the 3′ RR (Ju et al., 2007). Studies show some evidence that CTCF binding sites in the 3′ RR have some insulation activity (gray box) that is detected to impact as far as the “*hole*” gene. However, CTCF sites associated with downstream genes appear to provide an over-riding local influence (as discussed in the text).

As a potential “end” of B cell-specific regulation of the *Igh* locus, how might the 3′ RR help to focus DNA rearrangement events on the *Igh* locus and prevent inherently mutagenic events like DNA rearrangements and mutations from encroaching into neighboring downstream genes? We predicted that the 3′ RR might house an insulator region with CTCF as a major functional contributor, similar to insulator regions found in other loci (Phillips and Corces, 2009; Amouyal, 2010; Yang and Corces, 2011). In fact, (and before the era of high-throughput genomic analyses), EMSA with recombinant CTCF on 50 consecutive overlapping DNA fragments identified multiple CTCF sites (Garrett et al., 2005). These were associated with additional DNase I hs sites, hs5, 6, and 7, and with a segment 4 kb downstream of hs7, which because it is located 38 kb from the beginning of the 3′ RR has been termed “38.” Hs5 and hs7 were shown to confer insulator activity in a cell line assay (Garrett et al., 2005). Analysis of EMSA with nuclear extracts from B cell lines using supershift studies with specific antibodies for CTCF and Pax5 showed that this entire hs5–7-“38” region contained interspersed CTCF- and Pax5-binding sites (Chatterjee et al., 2011). Because Pax5 is a regulator of 3′ RR enhancers, these data suggested that Pax5 might help coordinate the actions of the enhancer-containing region with the insulator region. Here we describe studies of the contribution of the 3′ RR to *Igh* regulation throughout B cell development; in levels of H chain expression in plasma cells, in GT and CSR in B cells, and in use of V_H_ genes in VDJ joining in pro- and pre-B cells. For the most part, these involve the formation of loops associating components of the 3′ RR with target *Igh* sequences, as described in the following sections and as schematized in **Figure 2**.

## THE 3′ RR AFFECTS H CHAIN EXPRESSION IN PLASMA CELLS THROUGH CONTACTS WITH *Igh* TARGET SEQUENCES

A role for the 3′ RR in high levels of *Igh* expression in plasma cells was inferred when we established that the entire 3′ RR was deleted in a mouse plasma cell line that had lost 90% of its *Igh* expression levels (Gregor and Morrison, 1986; Michaelson et al., 1995). That the 3′ RR could loop to engage target *Igh* sequences was predicted from studies of another mouse plasma cell line, in which we detected an inversion of a segment extending from expressed V_H_ gene sequences to the 3′ RR palindromic region (Calvo et al., 1991; Ju et al., 2007). Resolution of a loop formed by interactions between the V_H_ gene and the 3′ RR is the simplest intermediate to account for this inversion. Documentation of such a loop structure came upon implementation of the chromosome conformation capture (3C) method in a plasma cell line: here we showed physical interactions involving 3′ RR enhancers and its CTCF-binding region with the J_H_ sequence that was part of the expressed V_H_ gene. The adjacent Eμ sequences were not essential for this interaction (Ju et al., 2007). The contacts associated with chromatin loop formation were severely disrupted in a different MPC11 variant, whose expressed *Igh* gene had been rendered non-functional by substitution of the hs1.2 enhancer by the NeoR gene (Ju et al., 2007). These data implied that an intact 3′ RR was essential for H chain expression in plasma cells and that H chain expression depended on intact physical interaction in *cis* of the 3′ RR with the expressed V_H_ gene. An extension of these observations from cell lines to mice has derived from targeted deletion of the entire enhancer-containing region of the 3′ RR in mice, which has confirmed a critical role of the 3′ RR in promoting high levels of *Igh* expression in plasma cells (Vincent-Fabert et al., 2010).

Efforts to identify proteins that support loop formation and concomitant *Igh* expression used a loss-of-function strategy employing lentiviral-mediated shRNA directed against CTCF, Oct-2, and OBF-1/OCA-B (Ju et al., 2011) in the MPC11 plasma cell line. In no case did we see effects on *Igh* expression. We conclude that proteins other than those targeted were required to support H chain expression, or that residual levels of CTCF, Oct-2, and/or OBF-1/OCA-B remaining after the knock-down were sufficient, or that these factors act in a redundant fashion and that simultaneous knock down of multiple factors is required for a decrease of *Igh* chain expression.

## TARGETED DELETIONS OF 3′ RR ENHANCERS REVEAL THEIR INVOLVEMENT IN GT AND CSR

The impact of targeted deletions of 3′ RR enhancers in mouse by a number of investigators has revealed their importance for two successive steps of the CSR process, i.e., transcription through C_H_ switch regions, followed by CSR. Deletion of the hs3B and hs4 region of the 3′ RR reduced switching to all isotypes except IgG1 (Cogne et al., 1994; Manis et al., 1998). The contribution to GT and CSR of the I/switch regions and of the 3′ RR enhancers has been fully demonstrated (reviewed in (Cogne and Birshtein, 2004). 3C studies on mature B cells undergoing CSR revealed interactions between the 3′ RR and switch regions through which transcription occurs prior to CSR (Wuerffel et al., 2007). These interactions were severely reduced in B cells from mice in which 3′ RR enhancers hs3B and hs4 were deleted. These data supported the importance of loop interactions between the 3′ RR and its target switch sequences for CSR. The distances involved range from ∼15 to ∼150 kb.

3C experiments also revealed cytokine-responsive chromosomal conformation involving the 3′ RR during GT and CSR (Wuerffel et al., 2007; Yan et al., 2011). Cytokine treatments that fostered switching to a particular isotype not only stimulate transcription of switch sequences of that isotype by activating the I region promoter upstream of switch sequences, but also result in specific increased 3C interactions between the 3′ RR and the isotype-specific switch region. Interestingly, a double deletion of hs3A and hs3B generated by the Eckhardt laboratory had no effect on either transcription or CSR (Yan et al., 2011). However, we found that in this doubly deleted mouse, isotype-specific interactions between switch regions and the 3′ RR ordinarily enhanced by cytokines were already at a high level in resting B cells, and there was a concomitant increase in interactions between the remaining 3′ RR enhancers, hs1.2 and hs4. These observations suggested that hs3A and hs3B modulate a functional hs1.2-hs4 3′ RR enhancer unit (Yan et al., 2011).

In fact, GT and CSR are generally unaffected after individual deletions of each of the four 3′ RR enhancers, including hs1.2 and hs4 (Manis et al., 1998; Vincent-Fabert et al., 2009; Bebin et al., 2010; Dunnick et al., 2011). Interestingly, a distinctive (but similarly functional) enhancer unit remains after each individual enhancer deletion, e.g., hs1.2, hs3B, hs4 (when hs3A is deleted); hs3A, hs3B, hs4 (when hs1.2 is deleted) and so on. This implies considerable flexibility in the structure and function of the 3′ RR enhancer unit, a point that is addressed further below. In all, the essential role of 3′ RR enhancers in GT and CSR can be met by their multiple alternative functional interactions with each other and with target switch sequences; these influence isotype-specific switching in response to cytokine signaling.

## TARGETED DELETION OF 3′ RR CTCF BINDING SITES HS5–7

Our studies have shown that during GT and CSR, the multiple modules of the 3′ RR, i.e., enhancers and the CTCF-binding region hs5–7, interact with I/switch regions and with the Pax5 transcription factor. Pax5 (reviewed in (Cobaleda et al., 2007) is essential for B cell identity and, through reporter assays, was shown to play an important role in regulating murine 3′ RR enhancers (Singh and Birshtein, 1993, 1996). To determine the function of the CTCF-binding region, we generated hs5–7 KO mice (Volpi et al., 2012). B cells from hs5–7 KO mice showed essentially normal GT and CSR except for a modest increase in IgG1^+^ cells upon switching in culture. One possibility to account for these observations is that interactions of *Igh* sequences with the CTCF/Pax5-binding site-rich hs5–7 region are secondary to the role of the 3′ RR enhancers and are not essential during CSR. Another possibility is that the deletion did not eliminate all candidate CTCF-binding sites. In fact, ChIP/Seq data (Degner et al., 2009) showed that the hs5–7 KO left behind a limited number of CTCF sites in the 3′ RR region, and other CTCF sites associated with each non-*Igh* downstream gene (R. Casellas, personal communication). Potentially, even a fraction of CTCF sites in this region or other CTCF-interacting sites are sufficient for appropriate biological activity. Similarly, we had anticipated that a reduction in insulator activity resulting from deletion of a large group of CTCF sites from the 3′ RR would enable the upstream unaffected 3′ RR enhancers to promote expression of downstream, non-*Igh* genes. However, our studies revealed only a modest increase in expression of the nearest downstream gene, *Tmem121*, while further downstream genes were unaffected (Volpi et al., 2012). It appears that local regulation of downstream genes by their own CTCF sites provides a back-up mechanism to restrain inappropriately regulated activity of the *Igh* locus from inflicting damage on non-*Igh* genes.

## INFLUENCE OF 3′ RR CTCF-BINDING REGION ON VDJ JOINING

CTCF has been described as a “master weaver of the genome” (Phillips and Corces, 2009). Thousands of genomic CTCF sites have been mapped, including those within the *Igh* locus (Garrett et al., 2005; Degner et al., 2009, 2011). Moving upstream (3′ to 5′) of the high-density CTCF-binding region in the hs5–7 region of the 3′ RR past the C_H_ and J_H_ regions that are devoid of CTCF sites, the CTCF sites that are closest to the 3′ RR are two sites located 5′ of the most 5′ D_H_ gene; by 3C, these have been shown to interact with the 3′ RR. Functional inactivation of the two D-associated CTCF sites abrogated normal VDJ joining (Guo et al., 2011); as a result, they have been named intergenic control region 1 (IGCR1). These studies imply a role of CTCF in VDJ joining. In fact, functional inactivation of CTCF in pro-B cells by shRNA (Degner et al., 2011) resulted in an increased distance between the interacting 3′ RR and D_H_/CTCF sequences, i.e., a reduction in V_H_-locus contraction, and an increase in anti-sense transcription in D_H_ and V_H_ regions. To determine, therefore, whether 3′ RR CTCF sites that bind to D_H_/CTCF are critical for the role of D_H_/CTCF in VDJ joining, we assessed a mouse with a targeted deletion of CTCF binding sites in the hs5–7 region of the 3′ RR (Volpi et al., 2012). Here, we were surprised to find essentially normal levels of VDJ joining in hs5–7 KO pro- and pre-B cells, except for a detectable increase in DQ52-J_H_3 usage at multiple stages of B cell development. In addition, there was a modest, albeit statistically significant reduction in *Igh* locus contraction, and an increase by twofold over wild-type in the use of proximal V_H_7183 genes while distal V_H_J558 usage was unaffected. Notably, allelic exclusion was correctly maintained. Although these data uncover an effect of the 3′ RR-CTCF-binding region on the *Igh* locus when VDJ joining is occurring presumptively through interactions of this region with D_H_/CTCF, they also imply the presence of considerable backups for proper *Igh* regulation.

## Pax5 AND CTCF AS REGULATORS OF THE 3′ RR DURING CSR

### Pax5

As a step toward further understanding mechanisms that control the 3′ RR, we have identified transcription factors that regulate 3′ RR enhancer activity. Experiments showed that the four 3′ RR enhancers are regulated by a common set of transcription factors, namely Oct-binding proteins, NFκB, and Pax5 (Michaelson et al., 1996), which could synergize for concerted repression (Singh and Birshtein, 1996) or for concerted activation of 3′ RR enhancers (Michaelson et al., 1996). YY1 has also been implicated (Gordon et al., 2003). Importantly, Pax5 appears to regulate each of the 3′ RR enhancers as well as the CTCF-binding region. Using chromatin immunoprecipitation (ChIP), we found that as B cells are induced to switch by culture with LPS +/- IL4, Pax5 shifts in its association with modules of the 3′ RR (Chatterjee et al., 2011). In resting B cells, Pax5 binds predominantly to hs4. At 48 h when GT and switch region-3′ RR interactions are at a peak, Pax5 has shifted away from hs4 to bind to upstream enhancer (hs1.2) and downstream insulator (hs7) flanking sites. At 96 h, when CSR has been completed, Pax5 regains hs4 binding as seen in resting B cells. Regardless of whether switching to γ3 or γ2b occurred by stimulation with LPS, or to γ1 through stimulation by LPS + IL4, the Pax5 pattern of binding to the 3′ RR was similar.

When we compared B cells that successfully undergo sequential steps in switch recombination with those that are deficient in GT and/or CSR (Chatterjee et al., 2011), we found that the Pax5-binding pattern to the 3′ RR is mechanistically associated with CSR. For example, stimulation of NFκB p50^–/–^ cells for 48 h with LPS + IL4 shows deficiency in normal GT; accordingly, the Pax5 profile is different from normal B cells. Pax5 continues to bind to hs4 although acquiring binding to hs1.2. In cells stimulated with anti-IgM + IL4, which undergo normal GT but fail to switch, the Pax5-binding pattern at 48 h is like that of cells stimulated by LPS + /-IL4, but at 96 h, the pattern is disrupted. Collectively, these data suggest that dynamic changes in Pax5 binding to the 3′ RR are supported by an isotype-independent scaffold on which GT and CSR occur.

### CTCF

To determine whether changes in CTCF binding to the 3′ RR were similarly associated with CSR, we analyzed binding of CTCF and its cofactor cohesin, this latter consisting of multiple subunits, including Rad21 (Chatterjee et al., 2011). In contrast to changes in Pax5 binding, we found relatively stable interactions of CTCF with the high-density CTCF-binding region in hs5–7 and “38” throughout the steps in GT and CSR that occurred in cells cultured with LPS + /-IL4. Also as expected, together with CTCF, Rad21 bound preferentially to hs7 upon stimulation with either LPS + /-IL4 or with anti-IgM + IL4. However, in resting B cells and independent of CTCF, Rad21 additionally bound to hs1.2 at low levels, and then at substantially increased levels at 48 h of stimulation before binding at reduced levels again to hs1.2 at 96 h. A similar pattern of CTCF-independent Rad21 binding to hs1.2 was detected in cells stimulated with anti-IgM + IL4.

Collectively, these data showed that CTCF and cohesin binding to the 3′ RR, both to cognate CTCF sites and independent of known CTCF sites, appear to contribute to a framework for the 3′ RR, while Pax5 has dynamic interactions with its binding sites. We have proposed (Chatterjee et al., 2011) that the multiple Pax5-binding sites in 3′ RR enhancers could support a scaffold structure: various enhancer deletions or shifts in enhancer occupancy could take place, leaving behind varying constellations of functional Pax5 sites.

## REGULATION OF 3′ RR BY DNA METHYLATION

We predicted that the 3′ RR is subject to epigenetic regulation as it acquires its functional capability. The 3′ RR essentially can be divided into two regions under separate epigenetic control, the 5′ palindromic enhancers and the more 3′ hs4-“38” region. Beginning in pro-B cells, the hs4-“38” region is associated with marks of active chromatin (Garrett et al., 2005) and with DNA demethylation (Giambra et al., 2008), which appear to be set in place by expression of Pax5 and linker histone H1. The upstream palindromic enhancers – hs3A-hs1.2-hs3B – acquire both epigenetic marks in B and plasma cells (Giambra et al., 2008).

The two *Igh* alleles in the mouse 70Z/3 pre-B cell line (C57Bl/6-derived and DBA/J-derived) can be distinguished by their stage during VDJ joining, their association with a polymorphic DNA segment that is subject to DNA demethylation (Giambra et al., 2008), and by the formation of loops involving the 3′ RR (Ju et al., 2011). The expressed VDJ-joined, C57Bl/6-derived, allele is associated in *cis* with a 3′ RR containing a deletion of hs3A-hs1.2 (with no apparent impact on *Igh* expression; Saleque et al., 1999). The polymorphic region located between hs4 and hs5 on this allele is demethylated. In contrast, the unexpressed DJ-joined allele (DBA/J-derived) fails to undergo looping in *cis* with its intact 3′ RR, and the hs4-hs5 sequence remains methylated. These data reinforce the role of the 3′ RR in *cis*-regulation of the *Igh* locus and imply that DNA demethylation in the 3′ RR, looping and *Igh* VDJ rearrangement and expression may be associated.

Interestingly, B cells stimulated for GT and CSR do not reveal any significant changes in chromatin marks of the 3′ RR (Garrett et al., 2005). Instead, we have identified progressive DNA demethylation of the 3′ RR (Giambra et al., 2008) and (Giambra, V., in preparation). These observations suggest that in resting B cells prior to stimulation for CSR, the 3′ RR is poised in its chromatin profile. We predict that DNA demethylation is associated with architectural changes by which the 3′ RR influences GT, CSR, and high levels of *Igh* expression in plasma cells. These epigenetic alterations of the 3′ RR during B cell development are summarized in **Box 1**.

**BOX 1. Regulation of methylation and chromatin modifications of 3′ RR during B cell development**

1. In pro-B cells, the hs4 enhancer and the CTCF-binding region hs5-“38” are demethylated and show marks of active chromatin. These marks are retained during B cell development. In B and plasma cells, the palindromic enhancers hs3A-1.2-3B acquire both epigenetic marks.

2. A polymorphic region between hs4 and hs5 reveals demethylation specific for the expressed allele in pre-B cells.

3. The 3′ RR in resting B cells is mostly methylated. In B cells stimulated to undergo class switching, the 3′ RR becomes progressively demethylated with limited accompanying changes in chromatin marks.

## DO *Igh* DNA REPLICATION PATTERNS SPECIFY ANOTHER TERMINUS OF THE *Igh* LOCUS?

Various landmarks might demarcate functional termini for the *Igh* locus; (1) the distinctive cluster of CTCF sites in hs5–7 that is located downstream of the C_H_ part of the locus and (2) ∼20 kb further downstream, the nearest non-*Igh* downstream gene, *Tmem121,* i.e., *hole.* In collaborative studies (Michaelson et al., 1997; Ermakova et al., 1999; Zhou et al., 2002a,b, 2005), we identified a replication origin downstream of *Tmem121* that is also a candidate for a functional B cell-specific terminus of *Igh* regulation. These studies showed that the *Igh* locus had different temporal patterns of DNA replication in non-B cells and at various stages of B cell development. In non-B cells, an origin of replication was identified ∼11 kb downstream of *Tmem121,* which is ∼30 kb downstream of the hs5–7 region and ∼76–79 kb downstream of the Cα gene. DNA sequences downstream of this landmark all replicated early in S. Beginning at this origin and moving upstream, i.e., 3′ to 5′, the 500 kb region within which C_H_, J_H_, D_H_, and V_H_7183 sequences were located replicated progressively later in S. This was consistent with the absence of activated origins of replication in this region. Sequences further upstream of the 500 kb transition region all replicated late in S. However, in pro-B and pre-B cells, the temporal transition region was eliminated as the entire *Igh* locus replicated early in S, indicative of the firing of multiple origins that were otherwise latent in non-B cells. Hence, this origin-containing region downstream of *Tmem121* appeared to demarcate upstream sequences that are under B cell-specific *Igh* regulation from downstream sequences under non-*Igh* control. Notably, in mature B cells and plasma cells, the temporal transition region was again evident and the replication pattern was similar to that seen in non-B cells. The change in replication was paralleled by a change in location of the *Igh* locus from a position at the nuclear periphery in non-B cells to away from the nuclear periphery in pro- and pre-B cells, with resumption of a nuclear periphery location in B and plasma cells. Analysis of replication dynamics in a cell line in which the 3′ RR enhancer region had been deleted, leaving behind the CTCF/Pax5-binding region and further downstream sequences, showed no difference compared to wild-type plasma cells (Michaelson et al., 1997). While these findings showed that the 3′ RR enhancer region is not essential for the timing of replication of the *Igh* locus in plasma cells, inferences about the role of the CTCF/Pax5-binding region in this process are not possible.

## SUMMARY

Here we have discussed two major modules of the 3′ RR, which extends ∼40 kb beginning downstream of Cα. The 5′ 28 kb segment contains four enhancers, which, collectively, support GT, CSR, and high levels of IgH expression in plasma cells. The ∼10 kb 3′ segment contains a region of high-density CTCF- and Pax5-binding sites with insulator activity. During B cell development, the 3′ RR-its enhancers and CTCF-binding region – is involved, via loop formation, with various target *Igh* sequences. These include: (1) CTCF sites upstream of D_H_ that are essential for normal VDJ joining and allelic *Igh* expression in pre-B cells; (2) I/switch sequences required for GT and CSR in B cells, and c) J_H_ and Eμ, which support *Igh* expression in plasma cells. While 3′ RR enhancers are essential for GT and CSR, as demonstrated by targeted deletions, independent deletion of at least seven of an estimated nine CTCF sites in the 3′ RR resulted in only a mild phenotype (Volpi et al., 2012). We found essentially normal VDJ joining but with a slight decrease in V_H_-locus contraction, a twofold increase in usage of proximal V_H_7183 genes and an apparent increase in DQ52-J_H_3 usage. Steps in GT and CSR appeared generally indistinguishable from wild-type, as was the chromosomal architecture of the 3′ RR assessed by 3C. In all, we conclude that the CTCF-binding region is a nidus for physical interactions with *Igh* targets of important biological consequence. However, there must be many back-ups that provide functional compensation to CTCF. These back-ups may include local regulators, such as CTCF sites associated with other neighboring genes, or proteins other than CTCF and/or epigenetic regulators that terminate B cell-specific regulation of the *Igh* locus.

## ONGOING KEY QUESTIONS

Which proteins/nucleic acids/other molecules are essential for loop formation? Are there different kinds of structural and functional loops? What do loops do? Do they engage the *Igh* locus in particular subnuclear domains for DNA rearrangements, mutation, etc. during different stages of B cell development? How does the 3′ RR function? What mediates architectural interactions among the 3′ RR enhancers themselves and between the enhancer and CTCF-binding modules? How does loop formation in the *Igh* locus relate to loops in other loci? What specifically does CTCF contribute to the structure and function of the *Igh* locus?

## Conflict of Interest Statement

The author declares that the research was conducted in the absence of any commercial or financial relationships that could be construed as a potential conflict of interest.
